# A model on leader-member exchange, psychological empowerment and teamwork in esports teams

**DOI:** 10.3389/fpsyg.2025.1653752

**Published:** 2025-11-11

**Authors:** Orcun Kececi, Laurentiu-Gabriel Talaghir, Veli Onur Celik, Teodora Mihaela Iconomescu, Mustafa Can Koc, Gabriel Marian Manolache

**Affiliations:** 1 Faculty of Applied Sciences, Osmaniye Korkut Ata University, Osmaniye, Türkiye; 2 Faculty of Physical Education and Sport, Dunarea de Jos University of Galati, Galati, Romania; 3 Faculty of Sports Sciences, Eskişehir Technical University, Eskişehir, Türkiye; 4 Faculty of Sports Sciences, Istanbul Gelisim University, Istanbul, Türkiye

**Keywords:** esports, leader-member exchange, psychological empowerment, teamwork, structural equation model

## Abstract

**Purpose:**

The aim of this research is to explore how leader-member exchange (LMX) affects teamwork through the mediation of psychological empowerment and discusses the interaction between the concepts within the framework of a relational model. Unfortunately, the previous related studies in the literature are limited to the examination of paired relationships between concepts and these concepts have not yet been sufficiently dealt with in relation to esports contexts. Therefore, the present study aims to fill this gap by collecting data from various sources.

**Methods:**

The data of the study, which was conducted with 804 professional esports players from Türkiye, the USA and South Korea who were selected by employing the conventional sampling method, were collected via an online survey and analyzed through structural equation modeling.

**Results:**

The findings revealed meaningful effects of leader-member exchange on teamwork and this effect was partially mediated by psychological empowerment.

**Conclusion:**

These findings are consistent with the Social Exchange Theory, Inputs-Process-Outcomes Model and Positive Organizational Behavior Theory and account for the relationship between leader quality and team performance. The study also contributes to the literature in regards to esports leadership and offers practical suggestions to team coaches and directors.

## 1 Introduction

Esports has recently become a strategic field worth studying not only as a digital entertainment medium but also from the point of views of leadership, organizational behavior and professional team management issues. Just like in other traditional sports, performance in esports teams and relationships among team members, leadership styles and psychological processes are remarkably interrelated (Gisbert-Pérez et al., 2024). Therefore, leader-member exchanges (LMX) is a pivotal construct referring to the quality of the relationship established by team coaches with players and significantly affecting team dynamics. What lies behind the impact of LMX on organizational outcomes are mostly cognitive-motivational variables like psychological empowerment, which is closely associated with certain dimensions including team collaboration and harmony (Graen and Uhl-Bien, 1995).

In today’s noticeably digitalized organizational structures and, especially, in rapidly thriving sectors such as esports, there is a growing need to explore team dynamics and leadership processes more comprehensively so as to understand these issues better. The research dealing with similarities and differences between leadership in esports and traditional sports is quite limited in number (Lee and Schoenstedt, 2011; Ross and Fisackerly, 2023; Behlau et al., 2025; Yuan, 2024). In addition, the impact of leadership behaviors in esports on organizational processes and outcomes has not yet been sufficiently demonstrated scientifically. Considering coach–player relationships in esports teams, determining how leader–member exchange influences psychological empowerment and teamwork will help to address the gap in the literature. Therefore, it is a need to enrich the literature by defining the outcomes of leadership in esports at organizational level (Keçeci and Çelik, 2024). Accordingly, the present study aims to fill this gap in the literature by dealing with the relationships between leader-member exchange, psychological empowerment and teamwork within the framework of a holistic structural model. These three concepts were often examined independently in the previous studies and, thus, comprehensive account of causal relationships and mediating mechanisms were not provided in their findings. The findings of the present study allow the evaluation of paired relationships within the framework of a holistic model and present how teamwork as an organizational outcome of leadership in esports is shaped through the mediation of psychological empowerment.

The primary aim of this study is to test a structural model unveiling the impact of leader-member exchange between coach and players in esports teams on teamwork through the mediation of players’ psychological empowerment levels. Achieving this aim will provide an important contribution to the literature by enabling a deeper understanding of the role of leadership processes in esports on individual and team dynamics. Clarifying the relationships among the concepts included as variables in the research will offer guidance for both the development of theoretical models and practical applications in the field of esports. In this way, a scientific basis will be established for developing more effective strategies to define the outcomes of the leadership process in esports teams, specifically through the concepts of psychological empowerment and teamwork. To achieve the objective of the study, the following hypotheses will be tested: (1) Leader–member exchange significantly affects psychological empowerment. (2) Leader–member exchange significantly affects teamwork. (3) Psychological empowerment significantly affects teamwork. (4) Psychological empowerment plays a mediating role between leader–member exchange and teamwork.

### 1.1 Leader-member exchange

LMX, one of the most effectual approaches to leadership processes, argues that leaders establish relationships at different levels of quality with each follower (Graen and Uhl-Bien, 1995). These differences have considerable impacts on certain outcomes such as work performance, organizational commitment and citizenship behaviors (Gerstner and Day, 1997). LMX Theory has its grounds on Role Theory (Graen, 1976), Social Exchange Theory (SET) (Blau, 1964) and Norm of Reciprocity (Gouldner, 1960). Relationships develop in time through exchanges of trust and values (Dienesch and Liden, 1986; Sparrowe and Liden, 1997). As mentioned in the multi-dimensional measurement model, LMX is accounted for under four dimensions: affect, loyalty, contribution and professional respect. Here, affect refers to sincere relationships, loyalty to mutual trust, contribution to voluntary actions and professional respect to appreciation of job skills (Liden and Maslyn, 1998).

In the present study, LMX was used in order to explain the quality of the relationship between the coach and players in esports teams and to function as one of the determinants of team dynamics.

### 1.2 Psychological empowerment

Psychological empowerment is a cognitive construct comprising competency, meaning, self-determination and impact dimensions and enhancing intrinsic motivation (Spreitzer, 1995). It supports self-development and affects organizational outcomes such as performance, commitment and innovation. The concept was grounded by Thomas and Velthouse (1990) Cognitive Motivation Model, and structured under four dimensions (meaning, competence, self-determination, impact) by Spreitzer (1995). Similarly, Zimmerman (1995) suggested a multi-layered model by incorporating individual, exchange and behavioral levels. The dimensions of empowerment are as follows: Meaning: the match between job and personal values; Competency: Trust in one’s skills and achievement; Self-determination: Freedom in job management; Impact: Perception of being able to effective on job outcomes (Deci and Ryan, 1985).

Psychological empowerment was incorporated into the study to comprehend cognitive and emotional effects of leader-member exchanges on players and make a comprehensive analysis of the construct as a mechanism mediating the indirect contribution to teamwork.

### 1.3 Teamwork

Teamwork refers to a fundamental component increasing productivity and efficiency in modern organizations (Salas et al., 2015; Driskell et al., 2018). It is possible to define this concept as the action taken to establish a dynamic and mutually dependent interaction between two or more individuals towards a common goal (Salas et al., 2018). Teamwork involves the cultivation of a shared mental model, communication, coordination, trust and role clarity as well as labor division (Driskell et al., 2018; Marks et al., 2001). Although the concept plays a key role in many sectors characterized with actions taken as a group, its definition and how to measure it are still a debatable issue (Rasmussen and Jeppesen, 2006).

Teamwork, in the present study, was taken as the final organizational outcome to measure and model the impact of leadership and psychological empowerment on collaboration, coordination and harmony towards the shared goal among team members.

### 1.4 Pair-wise relationships between the concepts

The studies carried out in different organizational contexts report that LMX significantly affects psychological empowerment. High-quality LMX relationships help employees to feel that they have more control over tasks, their skills are appreciated well enough and job-related tasks are meaningful to them (Aryee and Chen, 2006). Thanks to such relationships, individuals feel more valuable, effective and in a more central position in the organization and achieve higher levels of psychological empowerment dimension including meaning, competency, self-determination and impact (Hill et al., 2014; Wang et al., 2016). In addition, mutual trust and supportive environment procured by LMX causes considerable increase in individuals’ participation in job processes, which in turn motivates them while performing tasks and affects job outcomes such as organizational commitment (Srivastava and Dhar, 2016). Similarly, Schermuly and Meyer (2016) found that high-quality LMX relationships improve psychological well-being of employees and this relationship has indirect effects mediated by psychological empowerment. In brief, the relationship between LMX and psychological empowerment is shaped depending on whether one feels more effective and valuable in his/her working environment, which in turn contributes to the enhancement of organizational performance (Kim and George, 2005; Hu et al., 2018). In the present study, SET was taken as the basis to exhibit the relationship between leader-member exchange and psychological empowerment in esports teams. Developed by Blau (1964), SETargues that interpersonal relationships develop within the framework of mutual benefit, trust, and commitment. According to this theory, people respond to agents who appreciate and support them in their relationships and are fair to them in their behaviors. When the theory is taken as a reference for esports, it can be said that leader-member exchange is the reflection of organizational leadership in SET. Leaders in high-quality LMX relationships (coaches) provide followers (esports players) with valuable resources including trust, knowledge, support and involvement in decision-making processes. Players, as a response to these supports, display higher quality task performance, commitment and self-efficacy (Graen and Uhl-Bien, 1995; Wayne et al., 1997).

The previous research reports that the effects of quality leader-member exchange on teamwork generate meaningful outcomes at both individual and team level. Thanks to high quality LMX relationships, employees receive more social and structural support from their managers/directors, which significantly fosters behavioral integration in the team by facilitating mutual support, collaboration and knowledge sharing among team members (Herdman et al., 2017; Du et al., 2022). These relationships enhance team efficiency by creating an atmosphere promoting mutual trust among team members, foster collaborative attitudes and encourage action taken towards common goals (Chen, 2010). In addition, LMX stimulates not only individual task performances but also structural interaction in the team by positively impacting overall creativity of the team and collective decision-making processes (Du et al., 2022). Similarly, Xerri et al. (2021) found that LMX enhances individual resources like psychological capital etc. and this impact is also indirect and mediated, to a large extent, by organizational resources including teamwork and in-service training. Accordingly, it can be concluded that LMX is a social exchange mechanism supporting teamwork and a determining factor for the sustainability of integration within the team even during the presence of varied LMX situations. The assumption that leader-member exchange has both direct and indirect impacts on teamwork has its ground in Input-Process-Output Model, which is a classical theoretical framework discussing teamwork as a multi-dimensional and dynamic structure. Proposed by McGrath (1964) for the first time, the model was later revised by Ilgen et al. (2005) as well as Kozlowski and Ilgen (2006) into a comprehensive structure commonly preferred while analyzing modern teams. In other words, it can be taken as a reference in the analyses of esports teams.

The related studies report that psychological empowerment level plays a determining role in the quality and effectiveness of teamwork. Especially, when team members feel competent, impactful, self-determined and meaningful, they assume more active roles, share their knowledge with others and develop collaborative behaviors (Muduli, 2017). Sense of empowerment at team level considerably contributes to the formation of high-performing teams by fostering attempts towards reaching common goals and collective participation (Rasmussen and Jeppesen, 2006). Psychological empowerment not only increases individual motivation but also positively affects a team’s agility, stamina and ability to innovate (Ghen et al., 2019). The study by Sigwela (2020), which reported findings based on structural equation model (SEM), also support this relationship since he found significant direct effects of psychological empowerment on both work attitude and team efficiency. Therefore, psychological empowerment is a pivotal variable enhancing not only one’s work experiences but also boosting interaction within the team, commitment and collective efficiency. In this study, the positive effects of psychological empowerment on teamwork were examined according to the principles of Positive Organizational Behavior Theory, which was introduced by Fred Luthans. Luthans (2002) emphasized the determinant role of positive psychological resources having psychology-based strong impacts on individual work performance, job satisfaction and organizational commitment. As for esports contexts, it is assumed that achievement and effective coordination might depend not only on technical and mechanical skills but also on psychological ones. Therefore, this theoretical framework provides an explanatory theoretical basis.

## 2 Method

### 2.1 Research model

The study used the relational survey model to determine the relationships between leader-member exchange, psychological empowerment of team members and teamwork in esports teams. SEM was preferred to examine structural relationships between the study-specific variables. This model is an appropriate and robust method for reliably testing the direct and indirect relationships among leader–member exchange, psychological empowerment, and teamwork in esports, controlling for measurement errors and statistically verifying the mediation mechanism.

### 2.2 Population and sampling

The population of the study is professional esports players who actively take part in esports tournaments worldwide. Conventional sampling method was preferred to access the individuals of this population. A total of 804 professional esports players who know English and play for esports teams in Türkiye, the United States of America and South Korea volunteered to participate in the study. Accordingly, the sampling of the study is sufficient in number for the statistical analyses. The study sample consisted of 804 participants with diverse demographic characteristics. In terms of country of residence, 364 participants (45.3%) were from Türkiye, 251 (31.2%) from the United States, and 189 (23.5%) from South Korea. Participants’ ages ranged from 18 to 28 years, with a mean of 23.4. Regarding gender, the sample was predominantly male, comprising 598 males (74.4%) and 206 females (25.6%). Esports-related experience varied between 1 and 11 years, with an average of 6.1 years. Considering game genres, 281 participants (35.0%) played first-person shooter (FPS) games, 297 (37.0%) focused on multiplayer online battle arena (MOBA) games, and 226 (28.0%) specialized in sports games.

### 2.3 Data collection instruments

Within the scope of the study, leader-member exchange was measured by using Multi-dimensional Leader-member Exchange Scale (LMX-MDM) developed by Liden and Maslyn (1998). This 12-item scale measures four key dimensions of exchange relationship between leader and employee: affect, loyalty, contribution and professional support. Comprehensive validity and construct validity analyses were performed, and high internal consistency coefficients were reported while developing the scale. LMX-MDM is a commonly used measurement tool in the literature since it allows researchers to understand multi-dimensional structure of leader-member exchange.

The other data collection instrument employed in the study is Psychological Empowerment Scale developed by Spreitzer (1995), which measured participants’ psychological empowerment levels. This 12-item scale consists of four dimensions: meaning, competence, self-determination and impact. Spreitzer (1995), in his study, performed confirmatory factor analysis and found that dimensions are, in fact, separate constructs; however, they represent psychological empowerment concept together. In the original study, the internal consistency coefficient of the total scale was determined as 0.72 and was considered reliable. The scale was developed on the ground of self-efficacy-based motivation theories and structured to measure empowerment levels of employees.

In order to determine teamwork competencies of the participants, the researchers used Teamwork Scale for Youth (TSY) developed by Lower et al. (2017). The purpose of this scale is to measure young individuals’ skills regarding team collaboration, assuming responsibilities in the task and communication within team. Consisting of 8 items, the scale was proven to be valid in time, and its factor structure was confirmed by confirmatory analyses. In the original study, the internal consistency coefficient of the total scale was determined as 0.88 and was considered reliable. It stands out among the similar scales since it is user-friendly and measures teamwork perception at individual level. Although the Teamwork Scale for Youth was originally designed for young athletes, it was adopted in this study due to its emphasis on generalizable dimensions of teamwork such as communication, collaboration, and shared responsibility, which are also critical in professional esports contexts. To confirm its applicability, a confirmatory factor analysis (CFA) was conducted, and the results demonstrated an acceptable model fit (χ^2^/df = 2.73, CFI = 0.94, TLI = 0.92, RMSEA = 0.059). Therefore, the scale was considered valid for the current sample of professional esports players.

To ensure cross-cultural equivalence, a multi-group confirmatory factor analysis (CFA) was conducted across the three national subsamples (Türkiye, USA, South Korea). Configural and metric invariance were established (ΔCFI < 0.01), indicating that the factor structures were consistent across cultural groups (Cheung and Rensvold, 2002).

### 2.4 Data collection process

The data for the purposes of the study were collected by administering an online survey prepared by using Google Forms platform. The survey form was open for a period of 4 months and was closed to access once a sufficient number of participants had been reached. The potential participants were sent the survey via various digital channels (e-mail, social media, online groups etc.). Since the online survey required all items to be completed before submission, there were no missing data in the final dataset.

The participation in the study was on a voluntary basis and each participant was asked to read and confirm consent form before they provided the responses for the online survey. The study was conducted in conformity with the ethical principles specified in Helsinki Declaration (World Medical Association, 2013).

This research is a cross-sectional study in terms of data collection. As all data were collected from the same participants through self-report questionnaires at a single time point, the potential for common method variance (CMV) was assessed. In addition, Harman’s single-factor test was conducted, and the first factor accounted for less than 40% of the total variance, indicating that CMV was not a serious concern.

### 2.5 Data analysis

The first step of the data analysis process was to determine the validity and reliability of the data set for the further analyses. First of all, skewness and kurtosis values were calculated to check whether the variables displayed normal distribution or not (Tabachnick and Fidell, 2007). When the data were found to have normal distribution, confirmatory factor analysis was performed to test the validity of the proposed model for measurement. Accordingly, to what extent the model matches the data set was evaluated by examining various fit indices; namely, Chi Square/ degree of freedom (df), comparative fit indices (NFI, TLI, IFI, CFI, RMSEA), absolute fit indices (GFI, AGFI) and residual-based fit indices (RMR) (Hu and Bentler, 1999; Hooper et al., 2008). Later, as suggested by Fornell and Larcker (1981) AVE (Average Variance Extracted) and CR (Composite Reliability) values were calculated. Finally, Cronbach’s Alpha (*α*) coefficients were used to measure internal consistency of the scales. A scale is considered reliable when a value is achieved higher than 0.70 (Tavakol and Dennick, 2011).

After the data were confirmed to be suitable for the analyses, structural equation was formed to test the proposed model. At this phase, path analysis was preferred since it is an acknowledged method to determine causal relationships among variables (Hoyle, 2012; Kline, 2016; Hair et al., 2019). In the SEM, t value (critical value) was taken so as to test whether path values were significant or not and t ≥ 1.96 values were accepted as a statistically significant value (Hair et al., 2019). The indirect effects and their mediating roles in the model were tested by using bootstrap mediation analysis as suggested by Preacher and Hayes (2004). The SEM developed within the scope of this study used Variance Accounted For (VAF) value to determine to what extent indirect impacts are accounted. According to a widely acknowledged category presented in the literature (Hair et al., 2014), VAF < 0.20 values indicate no mediation, 0.20 ≤ VAF < 0.80 values partial mediation and VAF ≥ 0.80 full mediation. To test the significance of the mediaton, the bootstrap method was applied with a 95% confidence interval and 5,000 resamples. In the bootstrap analysis, the statistical significance of the indirect effect was evaluated based on whether the 95% confidence interval. The model parameters were estimated using the Maximum Likelihood (ML) method in AMOS, which is suitable for normally distributed continuous data.

Given that the data were collected from players nested within teams, the independence of observations was assessed. However, due to the lack of team identifiers, a single-level SEM was conducted treating all constructs as perceived at the individual level, consistent with prior studies examining teamwork as an individual-level perception (Salas et al., 2015; Driskell et al., 2018). Future research should adopt multilevel modeling to capture nested data structures.”

## 3 Results

### 3.1 Validity and reliability

The skewness and kurtosis values for all the variables ranged at ±1.5 interval, which indicates that the data used in the analyses met normal distribution assumption according to the criteria suggested by Tabachnick and Fidell (2007). Therefore, the data displayed suitable distribution characteristics to perform the parametric analyses.

The next step following the confirmation of normal data distribution was to perform confirmatory factor analysis (CFA). Various fit indices were examined to evaluate the fitness of the data for the model. The findings revealed an acceptable fit level. Although Chi Square value was found to be significant, this value is known to be greatly affected by the sampling size (*χ*^2^ = 312.47, df = 110, *p* < 0.001). Therefore, *χ*^2^/df ratio, which is normalized Chi Square, was calculated as 2.84, which indicated an acceptable fit since it was lower than 5.

As for the comparative fit indices, the following calculations were obtained: NFI = 0.91, TLI = 0.92, IFI = 0.93 and CFI = 0.95. These values indicate a good fit since both values were higher than the threshold values suggested by Hu and Bentler (1999) and Hooper et al. (2008). RMSEA value was calculated as 0.062, which also shows an acceptable fit level since it was lower than 0.08. GFI and AGFI, which are absolute fit indices, were calculated as 0.88 and 0.86, respectively. Since both values were higher than the threshold value (0.85), the model had an absolute fit level. Finally, RMR, a residual-based fit index, was calculated as 0.047, which shows that the model had a suitable fit since this value was lower than 0.08. All these findings show that the measurement structure of the proposed model significantly and suitably fit the data set.

The construct validity of the model was tested by calculating, as suggested by Fornell and Larcker (1981), AVE (Average Variance Extracted) and CR (Composite Reliability) values. According to the results, AVE values ranged between 0.52 and 0.68, which indicated acceptable convergent validity since these values were higher than the threshold value (0.50) for all constructs. Likewise, CR values were found to be between 0.76 and 0.89, implying high level of internal constancy for all the constructs due to higher values than 0.60 threshold value. As suggested by these findings, the constructs in the model met the construct validity criteria for both convergent validity and composite reliability.

Cronbach’s Alpha (*α*) coefficients were calculated to determine the reliability of the scales used in the study. According to the findings, these values ranged between 0.78 and 0.93 for all the dimensions. As for the dimensions of LMX scale, the calculations were as follows: *α* = 0.81 for “affect,” *α* = 0.85 for “loyalty,” *α* = 0.88 for “contribution” and *α* = 0.90 for “professional respect.” Cronbach’s Alpha values for the dimension of psychological empowerment scale were found to be *α* = 0.83 for “meaning,” *α* = 0.86 for “competence,” *α* = 0.78 for “self-determination” and *α* = 0.93 for “impact.” The alpha value for teamwork scale was calculated as 0.89. Thus, all the scales in the study were accepted as reliable since they exceeded the threshold value (0.70) suggested by Nunnally and Bernstein (1994) and Tavakol and Dennick (2011). Moreover, *α* ≥ 0.90 values obtained for some dimensions indicate perfect internal consistency according to the classification proposed by George and Mallery (2003). In conclusion, all the scales used for the purposes of the present study were highly reliable for measurement (see Table 1).

**Table 1:** Direct effects within the path analysis.

Independent variable	Path	Dependent variable	*t*	Effect value
Affect (LMX)	→	Meaning (PE)	4.83	0.582
Affect (LMX)		Competence (PE)	1.42	–
Affect (LMX)		Self-determination (PE)	0.87	–
Affect (LMX)	→	Impact (PE)	5.11	0.603
Affect (LMX)	→	Teamwork (TW)	4.24	0.476
Loyalty (LMX)		Meaning (PE)	1.61	–
Loyalty (LMX)		Competence (PE)	1.20	–
Loyalty (LMX)	→	Self-determination (PE)	5.30	0.621
Loyalty (LMX)	→	Impact (PE)	4.65	0.554
Loyalty (LMX)		Teamwork (TW)	0.94	–
Contribution (LMX)	→	Meaning (PE)	5.72	0.668
Contribution (LMX)	→	Competence (PE)	5.93	0.695
Contribution (LMX)		Self-determination (PE)	1.75	–
Contribution (LMX)	→	Impact (PE)	5.44	0.639
Contribution (LMX)	→	Teamwork (TW)	4.98	0.591
Professional respect (LMX)	→	Meaning (PE)	6.17	0.713
Professional respect (LMX)	→	Competence (PE)	6.37	0.736
Professional respect (LMX)		Self-determination (PE)	1.38	–
Professional respect (LMX)	→	Impact (PE)	5.86	0.674
Professional respect (LMX)	→	Teamwork (TW)	5.25	0.617
Meaning (PE)	→	Teamwork (TW)	6.03	0.703
Competence (PE)	→	Teamwork (TW)	6.49	0.748
Self-determination (PE)		Teamwork (TW)	1.52	–
Impact (PE)	→	Teamwork (TW)	6.69	0.782

Dashes (−) indicate non-significant paths (*t* < 1.96). Only significant paths are reported with standardized coefficients.

### 3.2 Structural equation model

When the direct impacts analyzed within the SEM were examined, it was found that there were significant and positive relationships between various variables. The effect of *affect* on *meaning* was calculated as 0.582, on *impact* as 0.603 and on *teamwork* as 0.476, which indicates that affect has considerable impact on both individuals’ assigning meaning to their work and contribution to teamwork. The effect of *loyalty* on self-determination was found to be 0.621 and on *impact* as 0.554, which implies that loyalty level of the leader might improve individuals’ perceptions of self-determination and effectiveness in work. The impact of *contribution* on the following variables were also statistically significant respectively: *meaning* (0.668), *competence* (0.695), *impact* (0.639) and *teamwork* (0.591). This situation indicates that individuals’ contribution to tasks is a strong predictor of both psychological empowerment dimensions and teamwork. Similarly, the effects of *professional respect* on *meaning* (0.713), on *impact* (0.674) and *teamwork* (0.617) were high and significant, which shows that professional respect of the leader for a member plays a strong role in individual perceptions and contributions to team dynamics. Finally, *meaning*, a component of psychological empowerment, had a significant effect (0.703) on *teamwork* while the effect of *competence* was calculated as 0.748 and *impact* as 0.782. These findings support the fact that individuals’ assigning meaning to tasks, feeling competent and thinking that they are efficient in their work have a direct impact on teamwork (see Table 2).

**Table 2:** Mediating effects within the model.

Independent variable (leader-member exchange)	Mediator variable (psychological empowerment)	Dependent variable (teamwork)	Indirect effect	Total effect	VAF
Affect	Meaning	Teamwork	0.409	0.885	0.462
Contribution	Meaning	Teamwork	0.468	1.059	0.442
Professional respect	Meaning	Teamwork	0.501	1.118	0.448
Contribution	Competence	Teamwork	0.519	1.110	0.467
Professional respect	Competence	Teamwork	0.551	1.168	0.472
Affect	Impact	Teamwork	0.472	0.948	0.498
Contribution	Impact	Teamwork	0.500	1.091	0.458
Professional respect	Impact	Teamwork	0.527	1.144	0.461

The examination of indirect impacts within the scope of the model revealed significant impacts of mediating variables in the relationships between independent variables and teamwork. Mediation impacts were determined by calculating VAF values. Accordingly, *meaning* variable played a partial mediating role in the relationships between *teamwork* and *affect* (VAF:0.462), *contribution* (VAF:0.442) and also *professional respect* (VAF: 0.448). Similarly, *competence* variable partially mediated in the relationships between *teamwork* and *contribution* (VAF:0.467), and *professional respect* (VAF: 0.472). Also, the effects of *contribution* (VAF = 0.458) and *professional respect* (VAF = 0.461) on *teamwork* through *impact* (VAF: 0.498) were also partial. These findings show that both leader-member exchange dimensions and psychological empowerment components contribute to teamwork and this contribution is often enhanced through indirect interventions as well. Therefore, we can conclude that meaning, competence and efficiency perceptions are effective mechanisms in transforming leadership-based perceptions into team behavior.

Table 3 presents the standardized direct effects with their standard errors, *t*-values, *p*-values, and 95% bias-corrected confidence intervals. All paths with *t* ≥ 1.96 were considered statistically significant at the 0.05 level. Also, the table reports the bootstrapped indirect effect obtained from 5,000 resamples with BCa 95% confidence intervals.”

**Table 3:** Path analysis based on scale totals.

Path	*β* (Std.)	SE	*t*	*p*	95% BCa CI
LMX → PE	0.655	0.041	15.0	< 0.01	0.579–0.723
PE → TW	0.741	0.038	19.0	< 0.01	0.666–0.808
LMX → TW (direct)	0.563	0.044	12.0	< 0.01	0.471–0.641
LMX → TW (indirect)	0.485	0.040	12.0	< 0.01	0.408–0.566

*β* (Std.), Standardized Estimate; SE, Standard Error; *t*, Test Statistic; *p*, Probability Value; 95% BCa CI, 95% Bias-Corrected and Accelerated Confidence Interval.

The relationships between leader-member exchange, psychological empowerment and teamwork were analyzed at uni-dimensional construct level. The findings obtained showed that the direct impact of leader-member exchange on psychological empowerment was 0.655, the direct impact of psychological empowerment on teamwork was 0.741 and the direct impact of leader-member exchange on teamwork was 0.563.

Under the light of these calculated values, indirect impact of leader-member exchange on teamwork was found to be 0.485 through the mediation of psychological empowerment, which amounts to 1.048 together with the impact mentioned above. The corresponding VAF value was also calculated to be 0.463, which indicates a partial impact. Accordingly, the effect of leader-member exchange on teamwork was significant not only directly but also indirectly through the mediation of psychological empowerment. Although one of the total effect coefficients exceeded 1.0, this was due to the use of unstandardized estimates and the additive nature of direct and indirect paths. A multicollinearity check indicated no critical overlap among predictors (VIF < 5), suggesting that the model specification remained acceptable.

**Figure 1:**
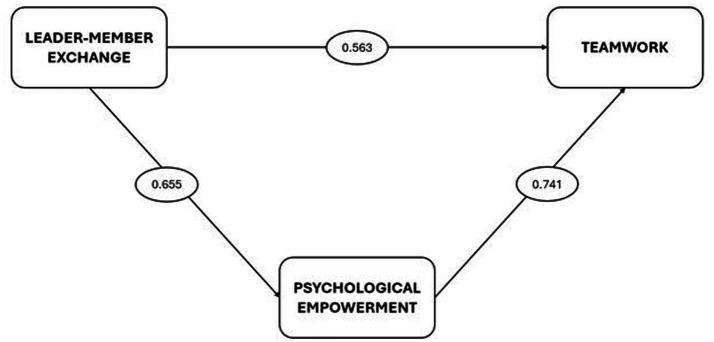
Paths and magnitude of significant values based on the dimensions in the model.

When Figure 1 is examined, the analysis results indicate that leader–member exchange significantly enhances players’ levels of psychological empowerment, and this effect is found to be both a direct and indirect determinant of teamwork. In other words, high-quality relationships between coaches and players foster a greater sense of competence, meaning, and impact among players, which in turn strengthens communication, coordination, and collective goal orientation within the team. Therefore, leader–member exchange emerges as a central factor that supports team cohesion and collective performance through the mediating role of psychological empowerment.

## 4 Conclusion

### 4.1 Discussion

The purpose of this study is to develop a model by dealing with leader-member exchange as an input and psychological empowerment as an organizational process and teamwork as an organizational outcome. This model exhibits the effects of leader-member exchanges on individuals’ psychological empowerment levels and claims that teamwork is not only a structural but also a psychology-based process. The inclusion of psychological empowerment in the model as a mediating variable enables researchers to have a deeper understanding of the effects of leader behaviors on employees’ subjective experiences. The contribution of this study to the literature is to allow, at conceptual and empirical level, a better grasp of how leadership processes and team collaboration are shaped within esport-specific team dynamics. Moreover, the study aims to achieve practical outcomes that are likely to offer suggestions applicable to coaches and leaders in addition to its theoretical contribution to the literature. This study also has the potential to become a valuable resource to understand which coach leadership behaviors enhance psychological empowerment of players and how this enhancement impacts team collaboration. The SEM developed within the scope of this study revealed that leader-member exchange has a high-level significant effect on players’ psychological empowerment. This finding shows that the quality of one-on-one relationship between coach and players has a direct effect on meaning assigned by players to the tasks, their confidence on their competencies, impacts on tasks and their perception of self-determination. In accordance with the cognitive-motivational explanations of Spreitzer’s (1995) psychological empowerment model, the study confirms that leadership exchanges trigger individuals’ internal motivational resources.

The findings obtained showed that the direct impact of leader-member exchange on teamwork is also statistically significant, which indicates that certain dimensions such as affect between coach and players, mutual loyalty, contribution expectation and professional respect are closely related to behavioral outcomes including team collaboration, common goal orientation and collective coordination. In addition, the SEM revealed that psychological empowerment has a medium-level effect on teamwork; a certain amount of the effect of leader-member exchange on teamwork is indirectly realized via this variable, which signifies a strong impact mechanism that operates both directly and indirectly. These findings are also significant within the framework of SET (Blau, 1964). It is clear from the findings that supportive and fair relationships established by coaches considerably encourage voluntary attempts of players and foster their feelings of belonging and team cohesion (Graen and Uhl-Bien, 1995; Wayne et al., 1997), which supports the findings of the present study and are consistent with the previous research emphasizing direct and mediating effects of leader-member exchange on positive organizational outcomes (Hill et al., 2014; Wang et al., 2016; Srivastava and Dhar, 2016; Spreitzer, 1996). Accordingly, positive behaviors perceived by players throughout leader-member exchange in esports teams have positive effects on teamwork, which is a study-specific organizational outcome in the present research. In addition, the findings obtained from the analyses of each dimension of psychological empowerment are quite striking. Especially meaning, competence and impact dimensions have a high-level effect on teamwork and strong relationships with leader-member exchange dimensions. The fact that affect, contribution and professional respect dimensions significantly predict these three dimensions of psychological empowerment reinforces mediating relationships more. In this context, theoretical explanations developed by researchers such as Zimmerman (1995) and Deci et al. (1989) regarding empowerment components were empirically confirmed with the findings of the present study. Thus, when IPO model was taken as a reference, psychological empowerment actions taken by leaders, which refer to “processes” variable in the model, is a unity of meaningful behaviors affecting outcomes.

The findings of the study are noticeably consistent with those obtained from the previous studies dealing with the relationships between leader-member exchange, psychological empowerment and teamwork. The study conducted by Aryee and Chen (2006) in Chinese context found that the effect of leader-member exchange on psychological empowerment contributes to organizational outcomes through job satisfaction and task performance and psychological empowerment plays a full mediating role in the process. At this point, partial meditation reported in the present study shows that leader-member exchange might affect teamwork also directly and function at a certain level without being dependent on psychological empowerment. This difference might be due to the fact that leader’s guiding effect are felt more directly in environments such as esports where individual tactic initiatives are more important. Hill et al. (2014) emphasized that the effect of leader-member exchange on psychological empowerment are more obvious in working environments characterized with intense electronic communication. Since esports, due to its nature, provides a context depending on digital and real-time communication might have resulted in more visible and functional exchange. Similarly, Srivastava and Dhar (2016) reported the effects of LMX and PE on extra role behaviors via organizational commitment and increased voluntary contribution levels of employees. This finding is valuable in that team members develop strategies together and actively contribute to decision-making processes in esports contexts. The findings obtained in this study are also consistent with those of the study conducted by Kim and George (2005) in service sector. They found a positive correlation between leader-member exchange and psychological empowerment and this relationship was consistent regardless of demographic differences. Similarly, the data collected from three different cultural contexts in our study (Türkiye, The USA and South Korea) also show that this relationship structure has an inter-contextual validity. The research carried out by Xerri et al. (2021) found that LMX improves personal psychological capital and this impact is reinforced by some resources such as teamwork and training. This finding is a strong support for LMX → PE → Teamwork model, which was also observed in our study. Direct and indirect effects of certain dimensions such as contribution and professional respect on both PE and teamwork are among the issues that have been studied inadequately in the literature.

Chen (2010), in his study conducted with amateur baseball coaches, claimed that LMX model is more effective than transformative leadership, which is still valid in esports environments where one-on-one coach-player relationships are critically important. A leader’s ability to establish emotional bond as well as his strategical guidance directly affect esports players’ team motivation. However, there are studies reporting complex and multi-layered structure of these relationships. For instance, Du et al. (2022) highlighted that negative dynamics such as jealousy, distrust and conflict might emerge in teams with high variation of LMX. Since our study was conducted with a group in which high-quality LMX relationships display a homogenous structure, such negative outcomes were not observed. Herdman et al. (2017) specified that the quality of the relationship established by a leader with one of his superiors might affect how LMX variation is perceived by team members. Therefore, further studies might focus on the effects of relationships established by esports coaches not only with players but also with organizational hierarchy on team dynamics.

The present study deals with coach-player relationship from the perspective of social change and is based on the assumption that these relationships shape team collaboration through psychological perceptions. Besides, the sampling was confined to three countries (Türkiye, the USA, South Korea) and cultural variables were not taken into consideration. In addition, the data were collected by employing the self-report technique, which is a limitation having the risk of personal bias. The constructs such as psychological empowerment might be measured objectively by employing multiple data resources (i.e coach evaluations, behavioral observations etc.) (Zimmerman, 1995). Finally, the study has a cross-sectional structure and causality claims are quite limited due to the lack of longitudinal analysis. If rapid technological developments in esports industry affect human relationships, researchers should keep in mind that such developments might also mean shorter validity duration for cross-sectional studies.

The study tried to answer two basic questions: (1) Does LMX significantly affect teamwork? (2) Does PE mediate this effect? The findings provided positive answers to both questions. The direct effect of LMX on teamwork and partial mediation of PE were found to be significant, which indicates that leadership-based variables generate outcomes through exchanges not only at individual level but also at team level.

When the findings of the present research are compared to those of the previous studies in the literature, we can conclude that the innovative contribution of this study is to integrate the relationships between LMX, PE and teamwork both at theoretical and empirical levels and structure them in an original context like esports. The study also combined some theoretical frameworks such as Positive Organizational Behavior (Luthans, 2002; Avey et al., 2008). IPO model (Salas et al., 2015; McGrath, 1964) and SET (Blau, 1964) into a holistic structure and tested this structure with valid data. Accordingly, it provided original contributions to the literature by explaining how interpersonal micro leadership exchanges might generate macro outcomes at team level.

### 4.2 Theoretical implications

There are some studies in the literature mainly dealing with esports coaching. To illustrate, Cho et al. (2022) highlight that esports coaches should not merely focus on performance and they should assume a specific role supporting mental, social and emotional well-being of players. Such an approach clearly depicts that high-quality relationships between coach and players significantly contribute to psychological empowerment by improving players’ perceptions of meaning, competency and impact. Similarly, Watson et al. (2024) explored the effects of leadership roles performed by coaches on team dynamics and player improvement and reported the critical role played by coaches in effective communication, generating motivation and fostering team harmony. The study conducted by Railsback and Caporusso (2019) emphasizes the significance of human factors in esports performance and provides a strong ground to have a clear understanding of how leadership exchanges and psychological empowerment affect players’ performances and team collaboration. Lee et al. (2025), in their observational study, found that esports coaching is not confined to merely providing players with technical information and it also manages cognitive, physical and emotional performance components of the process. This multi-dimensional coaching approach considerably contributes not only to players’ skills but also to the increase in their motivation, stress management and levels of well-beings. When the findings of the present study were compared to those of the studies mentioned above, we can conclude that leader-member exchanges are a strong predictor of psychological empowerment and this relationship directly or indirectly affects teamwork. Studying these constructs from a multi-dimensional perspective resulted in more comprehensive findings in terms of conceptual unity and revealed the mediating roles taken by the dimensions of psychological empowerment such as meaning, competence and impact. The results of the present study also support SET, IPO model and Positive Organizational Behavior Theory. Supportive behaviors of esports coaches assuming a leadership role trigger psychological empowerment of players assuming a follower role, which in turn improves effectiveness of team behaviors. Thus, it was possible to explain how leadership exchanges developing at interpersonal micro level evolve into macro outcomes at team level by using a tangible model.

### 4.3 Practical implications

The related literature presents practical suggestions regarding esports coaching. For instance, Cho et al. (2022) proposes that if esports coaches want to improve players’ wellbeing and performance, they should embrace a supportive and inclusive leadership approach that is sensitive to their individual needs. Adopting such a leadership style might lead to increased team collaboration and performance since coaches support players’ psychological empowerment. Similarly, Watson et al. (2022) emphasize that, in addition to be equipped with knowledge and skills required for effective leadership, coaches might positively affect overall achievement of the team by fostering relationships within the team. The study conducted by Railsback and Caporusso (2019), which focuses on human factors affecting esports players’ performance, exhibits the significance of leadership that takes individual characteristics and personal needs of players into consideration. Sabtan et al. (2022), in their study conducted with professional League of Legends coaches, report players’ attitudes, long training hours, high stress levels and physical health problems as the main challenges faced by coaches. In this respect, it is obvious that coaches should not only develop game strategies but also they should be competent enough in establishing mutual trust via individual relationships, providing psychological support and adopting flexible leadership styles. This condition signifies the importance of player-centered leadership approach and high-quality leadership-member exchanges so that it is possible to maintain harmony within team and sustainable achievement.

From the perspective of psychological science, the finding that psychological empowerment significantly mediates the effect of LMX on team collaboration aligns with fundamental mechanisms in the organizational behavior literature. Bektas and Sohrabifard (2013) emphasize that psychological empowerment processes, which strengthen employees’ perceptions of meaning, autonomy, competence, and impact, enhance the synergy of teamwork. In this perspective, LMX builds a relationship based on trust and support, reinforcing members’ sense of autonomy and meaning; this, in turn, enables empowered individuals to develop a stronger commitment to team goals and to engage in innovative collaboration. Likewise, Mathieu et al. (2017) point out that modern team theories explain team success particularly through dynamic processes and emergent emotional states, and are not independent of psychological factors. Rasmussen and Jeppesen (2006) also state that organizational behaviors within teams are strongly associated with psychological variables. These findings support the bridging role of psychological empowerment between LMX and teamwork.

The results of the current study present a number of implications in regards to practical applications for people holding a leadership position, especially esports coaches. In digital sports environments, which are characterized with high tempo and intensive cognitive load, the quality of individual relationships between the leader and team members has considerable direct effects not only on individual performance but also on cohesion and collaboration within the team. Therefore, it is essential for coaches to establish high-quality relationships fostering affect, loyalty, contributions and professional respect. Also, encouraging psychological empowerment (i.e assigning meaningful tasks, involving players in decision making processes, ensuring self-determination etc.) strengthens the quality of teamwork and contributes to sustainable achievement. Esports clubs and coach training programs should develop more conscious leadership strategies and form structures to improve individual motivation by taking the findings of this study into consideration.

### 4.4 Limitations and further research

The study was conducted only with a group of professional esports players in Türkiye, the USA and South Korea, which might be considered a limitation for the generalizability of study findings in terms of cultural issues. In addition, there is a risk of perceptual bias as the data were collected by using self-report technique. Subjective constructs including psychological empowerment can be measured objectively by collecting data form various resources: obtaining subordinates’ opinions about seniors, transforming physical and digital traces of subordinates into measurable data and asking independent raters to observe and code the collected data. Finally, causality between variables cannot be measured elaborately since cross-sectional data was employed to test the research model.

Although teamwork is inherently a team-level construct, it was analyzed as an individual-level perception in this study. This approach may introduce ecological fallacy risks by assuming independence among nested observations. Future research should test the model using multilevel SEM to distinguish between within-team and between-team effects.

A notable limitation of this study is that the teamwork construct was measured using the Teamwork Scale for Youth, which was originally developed for adolescent athletes. Although its content focuses on fundamental teamwork processes applicable across different age groups, its use with professional esports players may raise concerns regarding content validity. Future research should employ teamwork scales specifically validated for adult or elite sports samples to ensure a closer conceptual match with professional esports contexts.

The further studies might include comparative analyses by testing the model on different sectors, cultural contexts and age groups. Especially, the effect of leader-member exchange variation on perception of justice within the team and collective performance should be examined in detail. Moreover, longitudinal studies tracking how psychological empowerment process changes and evolves in time can allow researchers to grasp a more conclusive understanding of causal relations. The model can be enhanced through the inclusion of new variable such as team communication, decision-making mechanisms and mediating role of digital platforms when dynamic structure of esports teams is considered. In conclusion, the findings reported in the present study creates a well-established ground to develop more refined and contextualized theoretical models regarding leadership and motivation in team-based organizations. In addition, the use of big data and artificial intelligence-based monitoring techniques can contribute to quantitatively tracking real-time changes in leader–member exchange and psychological empowerment, as well as to developing predictive models. Studies that adopt cross-disciplinary approaches by integrating findings from sport psychology, organizational behavior, and game studies can strengthen the comprehensive testing of the conceptual model. Moreover, examining the impact of structural and cultural differences among various esports genres (e.g., MOBA, FPS, simulation racing, sport) will broaden the generalizability of the results. Conducting similar analyses on hybrid or complex team organizations outside of esports (e.g., remote-working technology teams, online creative communities) is also important for testing the universal validity of the model.

## Data Availability

The original contributions presented in the study are included in the article/supplementary material, further inquiries can be directed to the corresponding authors.

## References

[ref1] Aryee S. Chen Z. X. (2006). Leader–member exchange in a Chinese context: antecedents, the mediating role of psychological empowerment and outcomes. J. Bus. Res. 59, 793–801. doi: 10.1016/j.jbusres.2005.03.003

[ref2] Avey J. B. Luthans F. Jensen S. M. (2008). Psychological capital: a positive resource for combating employee stress and turnover. Hum. Resour. Manag. 48, 677–693. doi: 10.1002/hrm.20294, PMID: 41164814

[ref3] Behlau C. Dreiskaemper D. Strauss B. (2025). Team dynamics in esports and traditional sports: similarities and differences. Team Perform. Manag. Int. J. doi: 10.1108/TPM-08-2024-0092, PMID: 35579975

[ref4] Bektas C. Sohrabifard N. (2013). Terms of organizational psychology, personnel empowerment and team working: a case study. Procedia Soc. Behav. Sci. 82, 886–891. doi: 10.1016/j.sbspro.2013.06.366

[ref5] Blau P. M. (1964). Exchange and power in social life. Chicago: Wiley.

[ref6] Chen C. C. (2010). Leadership and teamwork paradigms: two models for baseball coaches. Soc. Behav. Personal. Int. J. 38, 1367–1376. doi: 10.2224/sbp.2010.38.10.1367

[ref7] Cheung G. W. Rensvold R. B. (2002). Evaluating goodness-of-fit indexes for testing measurement invariance. Struct. Equ. Model. Multidiscip. J. 9, 233–255. doi: 10.1207/S15328007SEM0902_5

[ref8] Cho Y. Cohen E. Freund A. Yip J. Lee J. H. (2022). “Coaching in esports” in Reset: critical perspectives on esports. eds. Apperley T. Parikka J. Kow K. (North American: Routledge), 65–76.

[ref9] Deci E. L. Ryan R. M. (1985). Intrinsic motivation and self-determination in human behavior. New York: Plenum Press.

[ref10] Deci E. L. Vallerand R. J. Pelletier L. G. Ryan R. M. (1989). Motivation and education: the self-determination perspective. Educ. Psychol. 26, 325–346. doi: 10.1207/s15326985ep2603&4_6

[ref11] Dienesch R. M. Liden R. C. (1986). Leader-member exchange model of leadership: a critique and further development. Acad. Manag. Rev. 11, 618–634. doi: 10.2307/258314

[ref12] Driskell J. E. Salas E. Driskell T. (2018). Foundations of teamwork and collaboration. Am. Psychol. 73, 334–348. doi: 10.1037/amp0000241, PMID: 29792452

[ref13] Du J. Lin X. Cai Y. Sun F. Amankwah-Amoah J. (2022). When teamwork works: examining the relationship between leader-member exchange differentiation and team creativity. Front. Psychol. 12:646514. doi: 10.3389/fpsyg.2021.646514, PMID: 35126217 PMC8815316

[ref14] Fornell C. Larcker D. F. (1981). Evaluating structural equation models with unobservable variables and measurement error. J. Mark. Res. 18, 39–50. doi: 10.1177/002224378101800104

[ref15] George D. Mallery P. (2003). SPSS for windows step by step: a simple guide and reference. 4th Edn. Boston: Allyn & Bacon.

[ref16] Gerstner C. R. Day D. V. (1997). Meta-analytic review of leader–member exchange theory: correlates and construct issues. J. Appl. Psychol. 82, 827–844. doi: 10.1037/0021-9010.82.6.827

[ref17] Ghen A. T. S. Abub N. Z. Sazali K. H. I. Belkhamza Z. (2019). The effect of empowerment and teamwork on employee productivity. Int. J. Innov. Creat. Change 6, 377–380.

[ref18] Gisbert-Pérez J. García-Naveira A. Martí-Vilar M. Acebes-Sánchez J. (2024). Key structure and processes in esports teams: a systematic review. Curr. Psychol. 43, 20355–20374. doi: 10.1007/s12144-024-05858-0

[ref19] Gouldner A. W. (1960). The norm of reciprocity: a preliminary statement. Am. Sociol. Rev. 25, 161–178. doi: 10.2307/2092623

[ref20] Graen G. B. (1976). “Role-making processes within complex organizations” in Handbook of industrial and organizational psychology. ed. Dunnette M. D. (Chicago, IL: Rand McNally), 1201–1245.

[ref21] Graen G. B. Uhl-Bien M. (1995). Relationship-based approach to leadership: development of leader–member exchange (LMX) theory of leadership over 25 years: applying a multi-level multi-domain perspective. Leadersh. Q. 6, 219–247. doi: 10.1016/1048-9843(95)90036-5

[ref22] Hair J. F. Black W. C. Babin B. J. Anderson R. E. (2019). Multivariate data analysis. 8th Edn. Andover, Hampshire, United Kingdom: Cengage Learning.

[ref23] Hair J. F. Hult G. T. M. Ringle C. M. Sarstedt M. (2014). A primer on partial least squares structural equation modeling (PLS-SEM). United States of America: SAGE Publications.

[ref24] Herdman A. O. Yang J. Arthur J. B. (2017). How does leader-member exchange disparity affect teamwork behavior and effectiveness in work groups? The moderating role of leader-leader exchange. J. Manag. 43, 1498–1523. doi: 10.1177/0149206314556315

[ref25] Hill N. S. Kang J. H. Seo M. G. (2014). The interactive effect of leader–member exchange and electronic communication on employee psychological empowerment and work outcomes. Leadersh. Q. 25, 772–783. doi: 10.1016/j.leaqua.2014.04.006

[ref26] Hooper D. Coughlan J. Mullen M. R. (2008). Structural equation modelling: guidelines for determining model fit. Electron. J. Bus. Res. Methods 6, 53–60.

[ref27] Hoyle R. H. (2012). “Path analysis and structural equation modeling with latent variables” in APA handbook of research methods in psychology: Vol. 2. Quantitative, qualitative, neuropsychological, and biological. ed. Cooper H. (American Psychological Association), 333–346.

[ref28] Hu L. T. Bentler P. M. (1999). Cutoff criteria for fit indexes in covariance structure analysis: conventional criteria versus new alternatives. Struct. Equ. Model. Multidiscip. J. 6, 1–55. doi: 10.1080/10705519909540118

[ref29] Hu Y. Zhu L. Zhou M. Li J. Maguire P. Sun H. . (2018). Exploring the influence of ethical leadership on voice behavior: how leader–member exchange, psychological safety and psychological empowerment influence employees’ willingness to speak out. Front. Psychol. 9:1718. doi: 10.3389/fpsyg.2018.01718, PMID: 30258392 PMC6143838

[ref30] Ilgen D. R. Hollenbeck J. R. Johnson M. Jundt D. (2005). Teams in organizations: from input-process-output models to IMOI models. Annu. Rev. Psychol. 56, 517–543. doi: 10.1146/annurev.psych.56.091103.070250, PMID: 15709945

[ref31] Keçeci O. Çelik V. O. (2024). A qualitative study on e-sports players’ leadership perceptions regarding their team coaches. Pamukkale J. Sport Sci. 15, 144–166. doi: 10.54141/psbd.1380150

[ref32] Kim B. George R. T. (2005). The relationship between leader–member exchange (LMX) and psychological empowerment: a quick casual restaurant employee correlation study. J. Hosp. Tour. Res. 29, 468–483. doi: 10.1177/1096348005276498

[ref33] Kline R. B. (2016). Principles and practice of structural equation modeling. 4th Edn. New York: The Guilford Press.

[ref34] Kozlowski S. W. J. Ilgen D. R. (2006). Enhancing the effectiveness of work groups and teams. Psychol. Sci. Public Interest 7, 77–124. doi: 10.1111/j.1529-1006.2006.00030.x, PMID: 26158912

[ref35] Lee H. Kleinman E. Kim N. Park S. Harteveld C. Lee B. (2025). Crafting champions: an observation study of esports coaching processes. Proceedings of the CHI conference on human factors in computing systems (CHI ‘25), Yokohama, Japan.

[ref36] Lee D. Schoenstedt L. J. (2011). Comparison of eSports and traditional sports consumption motives. ICHPER-SD J. Res. 6, 39–44.

[ref37] Liden R. C. Maslyn J. M. (1998). Multidimensionality of leader-member exchange: an empirical assessment through scale development. J. Manag. 24, 43–72. doi: 10.1177/014920639802400105, PMID: 41132823

[ref38] Lower L. M. Newman T. J. Anderson-Butcher D. (2017). Validity and reliability of the teamwork scale for youth. Res. Soc. Work. Pract. 27, 716–725. doi: 10.1177/1049731515589614

[ref39] Luthans F. (2002). Positive organizational behavior: developing and managing psychological strengths. Acad. Manag. Exec. 16, 57–72. doi: 10.5465/ame.2002.6640181, PMID: 38191495

[ref40] Marks M. A. Mathieu J. E. Zaccaro S. J. (2001). A temporally based framework and taxonomy of team processes. Acad. Manag. Rev. 26, 356–376. doi: 10.5465/amr.2001.4845785

[ref41] Mathieu J. E. Hollenbeck J. R. van Knippenberg D. Ilgen D. R. (2017). A century of work teams in the journal of applied psychology. J. Appl. Psychol. 102, 452–467. doi: 10.1037/apl0000128, PMID: 28150984

[ref42] McGrath J. E. (1964). Social psychology: a brief introduction. New York: Holt, Rinehart & Winston.

[ref43] Muduli A. (2017). Workforce agility: examining the role of organizational practices and psychological empowerment. Glob. Bus. Organ. Excell. 36, 46–56. doi: 10.1002/joe.21800

[ref44] Nunnally J. C. Bernstein I. H. (1994). Psychometric theory. 3rd Edn. New York: McGraw-Hill.

[ref45] Preacher K. J. Hayes A. F. (2004). SPSS and SAS procedures for estimating indirect effects in simple mediation models. Behav. Res. Methods Instrum. Comput. 36, 717–731. doi: 10.3758/BF03206553, PMID: 15641418

[ref46] Railsback D. Caporusso N. (2019). Investigating the human factors in eSports performance. In Advances in human factors in wearable technologies and game design: Proceedings of the AHFE 2018 international conferences on human factors and wearable technologies, and human factors in game design and virtual environments, (pp. 325–334). Orlando, Florida, USA: Springer International Publishing.

[ref47] Rasmussen T. H. Jeppesen H. J. (2006). Teamwork and associated psychological factors: a review. Work Stress 20, 105–128. doi: 10.1080/02678370600920262

[ref48] Ross W. J. Fisackerly W. (2023). Do we need esports ecology? Comparisons of environmental impacts between traditional sport and esports. J. Electr. Gaming Esports 1. doi: 10.1123/jege.2022-0030

[ref49] Sabtan B. Cao S. Paul N. (2022). Current practice and challenges in coaching Esports players: an interview study with league of legends professional team coaches. Entertain. Comput. 42:100481. doi: 10.1016/j.entcom.2022.100481

[ref50] Salas E. Reyes D. L. McDaniel S. H. (2018). The science of teamwork: Progress, reflections, and the road ahead. Am. Psychol. 73, 593–600. doi: 10.1037/amp0000334, PMID: 29792470

[ref51] Salas E. Shuffler M. L. Thayer A. L. Bedwell W. L. Lazzara E. H. (2015). Understanding and improving teamwork in organizations: a scientifically based practical guide. Hum. Resour. Manag. 54, 599–622. doi: 10.1002/hrm.21628

[ref52] Schermuly C. C. Meyer B. (2016). Good relationships at work: the effects of leader–member exchange and team–member exchange on psychological empowerment, emotional exhaustion, and depression. J. Organ. Behav. 37, 673–691. doi: 10.1002/job.2060

[ref53] Sigwela T. (2020). The relationship between authentic leadership, psychological empowerment, work engagement and team effectiveness (Master’s thesis,: Stellenbosch University.

[ref54] Sparrowe R. T. Liden R. C. (1997). Process and structure in leader-member exchange. Acad. Manag. Rev. 22, 522–552. doi: 10.2307/259332

[ref55] Spreitzer G. M. (1995). Psychological empowerment in the workplace: dimensions, measurement, and validation. Acad. Manag. J. 38, 1442–1465. doi: 10.2307/256865

[ref56] Spreitzer G. M. (1996). Social structural characteristics of psychological empowerment. Acad. Manag. J. 39, 483–504. doi: 10.2307/256789

[ref57] Srivastava A. P. Dhar R. L. (2016). Impact of leader member exchange, human resource management practices and psychological empowerment on extra role performances: the mediating role of organisational commitment. Int. J. Product. Perform. Manag. 65, 351–377. doi: 10.1108/IJPPM-01-2014-0009

[ref58] Tabachnick B. G. Fidell L. S. (2007). Experimental designs using ANOVA, vol. 724. Duxbury: Thomson/Brooks/Cole.

[ref59] Tavakol M. Dennick R. (2011). Making sense of Cronbach's alpha. Int. J. Med. Educ. 2, 53–55. doi: 10.5116/ijme.4dfb.8dfd, PMID: 28029643 PMC4205511

[ref60] Thomas K. W. Velthouse B. A. (1990). Cognitive elements of empowerment: an “interpretive” model of intrinsic task motivation. Acad. Manag. Rev. 15, 666–681. doi: 10.5465/amr.1990.4310926

[ref61] Wang D. Gan C. Wu C. (2016). LMX and employee voice: a moderated mediation model of psychological empowerment and role clarity. Pers. Rev. 45, 605–615. doi: 10.1108/PR-11-2014-0255

[ref62] Watson M. Jenny S. E. Johnson T. (2024). “Esports coaching” in The Routledge handbook of esports. eds. Freeman B. Funk M. S. McNally M. J. (London: Routledge), 318–332.

[ref63] Watson M. Smith D. Fenton J. Pedraza-Ramirez I. Laborde S. Cronin C. (2022). Introducing esports coaching to sport coaching (not as sport coaching). Sports Coach. Rev. 14, 263–282. doi: 10.1080/21640629.2022.2123960, PMID: 40989069

[ref64] Wayne S. J. Shore L. M. Liden R. C. (1997). Perceived organizational support and leader-member exchange: a social exchange perspective. Acad. Manag. J. 40, 82–111. doi: 10.5465/257021

[ref65] World Medical Association (2013). World medical association declaration of Helsinki: ethical principles for medical research involving human subjects. JAMA 310, 2191–2194. doi: 10.1001/jama.2013.281053, PMID: 24141714

[ref66] Xerri M. J. Farr-Wharton B. Brunetto Y. (2021). Nurturing psychological capital: an examination of organizational antecedents: the role of employee perceptions of teamwork, training opportunities and leader–member exchange. Pers. Rev. 50, 1854–1872. doi: 10.1108/PR-05-2019-0222

[ref67] Yuan S. (2024). The ecosystem of eSports and traditional sports: a comparative analysis. Asian J. Sport Hist. Cult. 3, 266–288. doi: 10.1080/27690148.2024.2380307

[ref68] Zimmerman M. A. (1995). Psychological empowerment: issues and illustrations. Am. J. Community Psychol. 23, 581–599. doi: 10.1007/BF02506983, PMID: 8851341

